# Meticillin-resistant *Staphylococcus aureus* Contact Screening Strategy in a Low Prevalence Setting; a Nested Case-Control Study^☆^

**DOI:** 10.1016/j.infpip.2022.100211

**Published:** 2022-02-25

**Authors:** Magi Bächli, Rami Sommerstein, Carlo Casanova, Sara Droz, Marianne Küffer, Jonas Marschall

**Affiliations:** a Department of Infectious Diseases, Bern University Hospital, University of Bern, Switzerland; b Institute for Infectious Diseases, University of Bern, Switzerland; c Division of Infectious Diseases, Department of Medicine, Washington University School of Medicine, St. Louis, MO, United States

**Keywords:** MRSA, Screening strategy, Spa type, Contact patient, Nosocomial transmission

## Abstract

**Background:**

The optimal screening strategy in hospitals to identify secondary cases after contact with a meticillin-resistant *Staphylococcus aureus* (MRSA) index patient in a low prevalence setting is not well defined. We aimed at identifying factors associated with documented MRSA transmissions.

**Method:**

Single center, retrospective, nested case-control study. We evaluated the screening strategy in our 950 bed tertiary care hospital from 2008 – 2014. Room and ward contacts of MRSA index patients present at time of MRSA identification were screened. We compared characteristics of *Staphylococcus aureus* Protein A (spa)-type matched contact patients (cases) to negative or spa-type mismatched contact patients (controls).

**Results:**

Among 270,000 inpatients from 2008 – 2014, 215 MRSA screenings yielded 3013 contact patients, and 6 (0.2%) spa-type matched pairs. We included 225 controls for the nested case-control study. The contact type for the cases was more frequently “same room” and less frequently “same ward” compared with the controls (*P* = 0.001). Also, exposure time was longer for cases (median of 6 days [IQR 3–9]) than for controls (1 day [0–3], *P*=0.016).

**Conclusion:**

The extensive MRSA screening strategy revealed only few index/contact matches based on spa-typing. Prolonged exposure time and a shared room were significantly associated with MRSA transmission. A targeted screening strategy may be more useful in a low prevalence setting than screening entire wards.

## Introduction

The optimal screening strategy to identify secondary cases after contact with a meticillin-resistant *Staphylococcus aureus* (MRSA) index patient in a low prevalence setting is not well defined. There is evidence that targeted screenings of high risk patients (such as contact patients) are less cost-intensive than universal MRSA screening strategies [1,2]. Furthermore, screening strategies can contribute to the success of a MRSA prevention strategy and lead to a reduction of nosocomial MRSA infection rates [3,4]. Harbarth *et al.* reported that a reasonable approach in most European hospitals with MRSA on-admission prevalence of <5% is to apply targeted rather than universal screening, taking the local MRSA epidemiology, infection control practices and vulnerability of the patient population into consideration [5].

The German Robert Koch Institute recommends MRSA screening for those patients who had contact to MRSA patients during an inpatient stay (e.g. in the same room), however, without specifying details [3]. The US Centers for Disease Control and Prevention note that decisions about targeted surveillance should be made in the context of the local incidence and prevalence of colonization with multi-drug resistant organisms [6]. Despite only moderate evidence, the Society for Healthcare Epidemiology of America recommends implementing an active MRSA surveillance testing as part of a multifaceted strategy to control and prevent MRSA [7]. The optimal timing of roommate post-exposure screening as well as the interval of a repeated sampling are neither described in national nor in international guidelines [3,4]. Two studies suggest a testing interval of approximately 1 week [11,12]. Accordingly, optimal timing of roommate post-exposure screening remains uncertain.

The extensive screening strategy in our 950 bed tertiary care hospital consisted of screening contact patients who shared room or ward with a confirmed MRSA index patient by means of swab cultures. Screening included nasal and inguinal swabs as well as wound (if available) and urine samples (in case of an indwelling urinary catheter). This policy was consistent with Harbarth *et al.*, who suggested that screening of multiple body sites provides better diagnostic accuracy data and improves rates of detection, however, when screening multiple body sites, nasal screening was revealed to offer the highest yield for detection [5]. We suspected that transmissions are associated with distinct temporal and spatial factors and that an extensive screening strategy in a low prevalence setting such as ours is not necessary.

## Methods

### Study design and setting

A single center retrospective cohort with a nested case control study from 2008-2014 was performed at Bern University Hospital, a 950 bed tertiary care hospital in Bern, Switzerland. All patients hospitalized in the period 2008–2014 were included.

For the cohort study, eligibility criteria for an *index* patient were hospitalization and MRSA detection (from clinical specimens), documented by a positive culture from the microbiology laboratory.

Characteristics of index and contact patients from 2008-2014 were collected in an Excel spreadsheet (Microsoft Office®) as part of our routine. There were no study-specific interventions administered.

*Contact* patients were determined using the patient administration system (SAP®; SAP SE, Walldorf, Germany). The screening strategy consisted of examining *contact* patients who shared room or ward with a proven MRSA *index* patient by nasal and inguinal swab cultures, plus wounds (if present) or urine samples (in case of an indwelling urinary catheter). Samples were processed with selective culture media (mannitol salt-agar-oxacillin biplates) and MRSA were confirmed according to standard laboratory procedures.

We traced back contacts during hospitalization in our hospital group up to one year before the index case was reported to us and did not define a minimal contact time (i.e. exposure). Those contact patients who had shared a room with an index patient and were discharged, were screened upon readmission up to one year following the exposure. For contact patients who were not re-admitted to our hospital group within a year, the indicator was deleted in the patient administration system.

For the nested case-control study, we considered all contacts from screenings that yielded a secondary MRSA case as “cases”. Negative or spa-type mismatched contact patients were included as “controls”. We collected gender, age, spatial factors as ward-type (according to medical specialty) and contact-type (same room [double or multiple bed], versus same ward), and temporal factors as exposure time and interval between most recent contact and screening from the electronic records. The spa type of all non-duplicate MRSA isolates at the institution during the study period was determined. DNA amplification and sequencing were performed using primers from the Ridom Spa Server website (https://spa.ridom.de). Spa types were determined using the Ridom StaphType software (Ridom, Münster, Germany) [8].

To address potential sources of bias, *index* patients as well as *contact* patients in whom MRSA was only detected by PCR and not confirmed with culture were not included. Contact patients who were not screened within one year of the exposure were not included.

### Statistics

In a descriptive analyse we compared characteristics of matched contact patients (cases) with clonal-mismatched or negative contact patients (controls) within the nested case control subcohort.

## Results

### Outcomes

**Figure 1:**
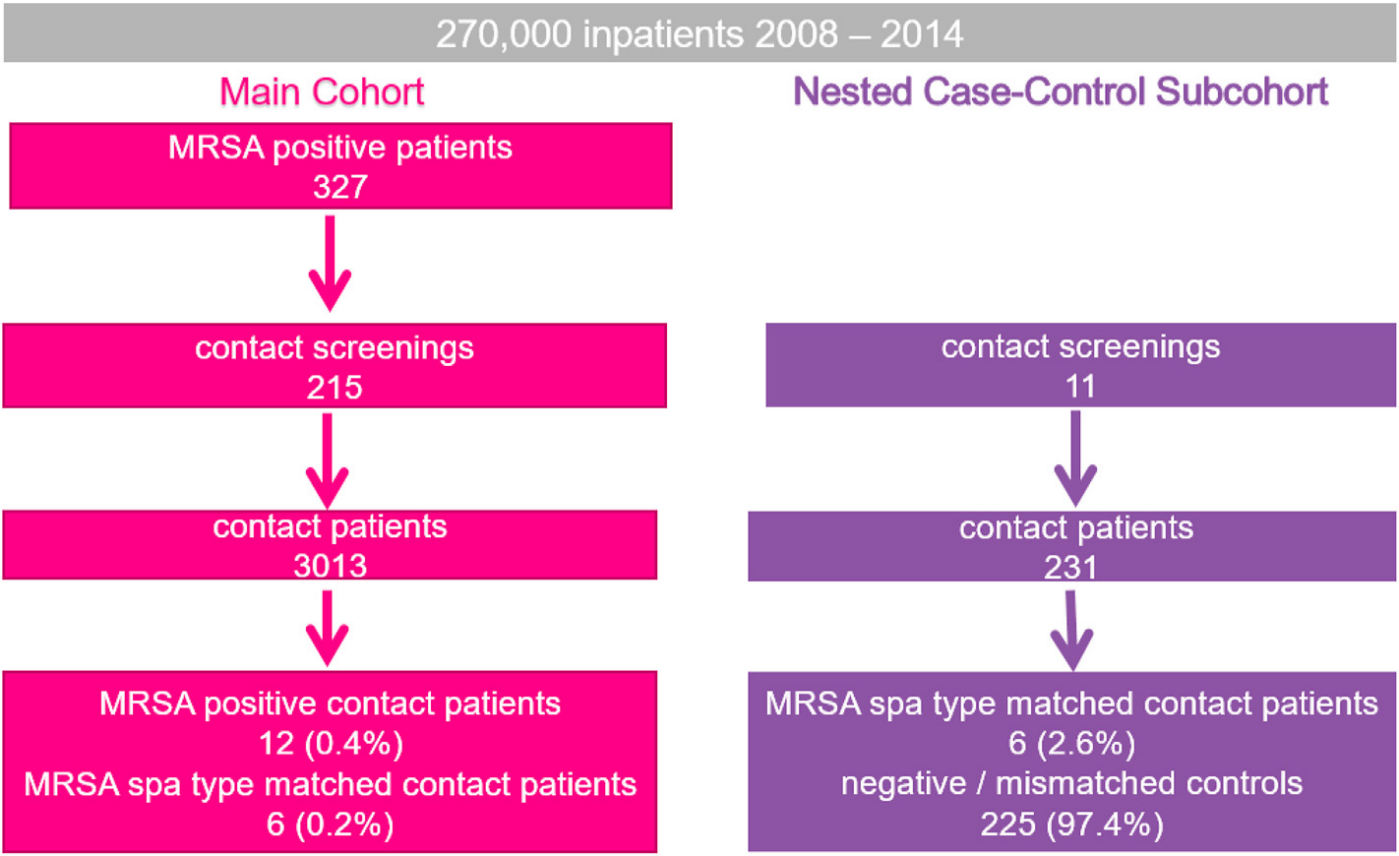
Flowchart Overview of patients with meticillin-resistant *Staphylococcus aureus* detection between 2008 and 2014. The flowchart of how Index/Contact spa-type matched patients were identified and the flowchart of the nested case-control subcohort is shown.

**Figure 2:**
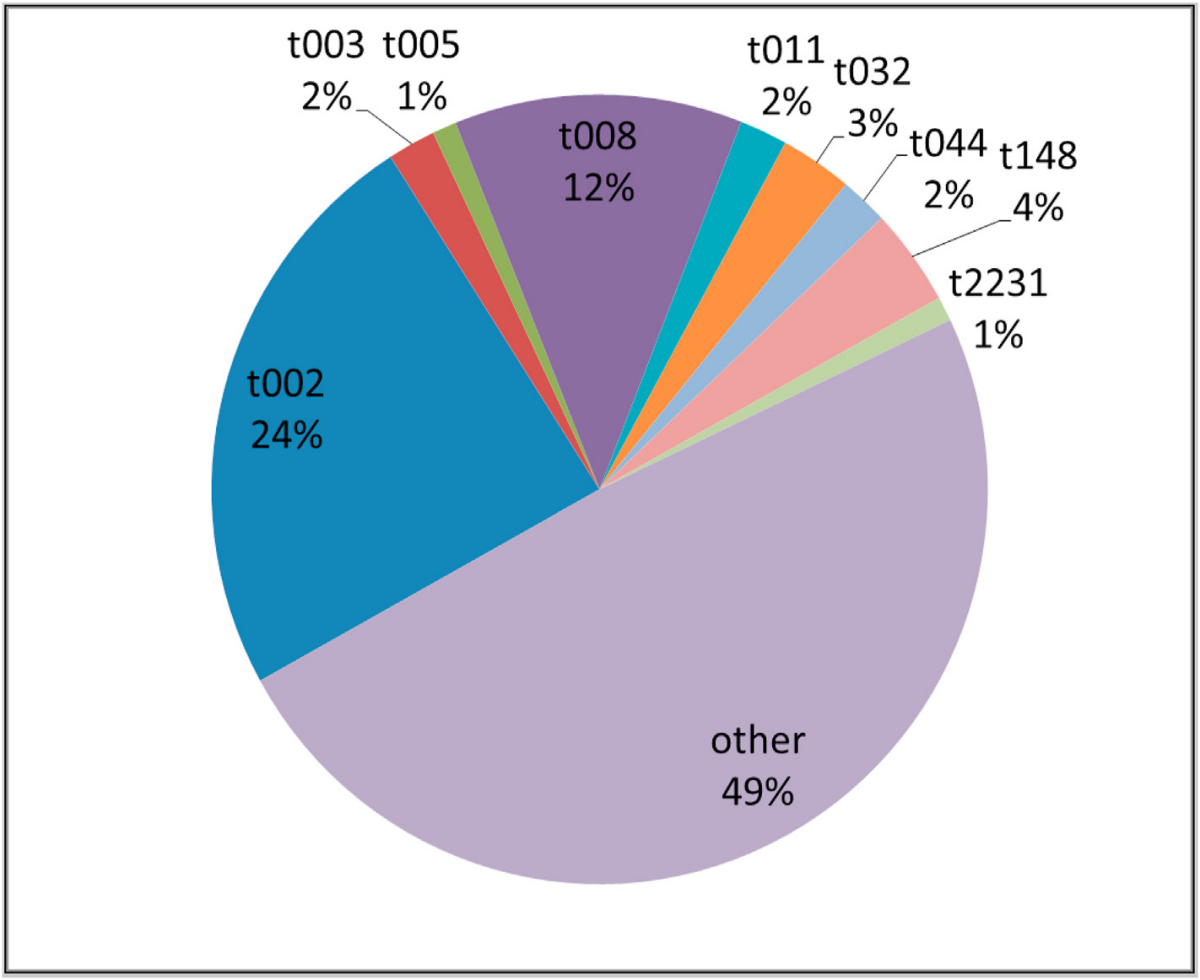
Spa-type frequencies of 339 non-duplicate MRSA Isolates between 2008 and 2014 at Bern University Hospital.

Out of 270,000 inpatients from 2008 – 2014, we identified 327 MRSA positive patients. In 215 of these cases, the newly-detected MRSA patient was not placed on contact isolation precautions in time. Therefore, 215 contact screenings of exposed patients were necessary, resulting in the screening of 3013 contact patients. Of these contact patients, 12 (0.4%) were MRSA positive and among them 6 (0.2%) had an identical I/C spa type (Figure 1). Therefore, we identified 339 non-duplicate MRSA in our institution (327 index patients plus 12 contact patients) and the predominant spa type we encountered was t002 (n=82; 24%) (Figure 2).

The nested case control subcohort revealed 231 contact patients out of 11 contact screenings. Among them, there were 6 (2.6%) MRSA spa type matched contact patients and 225 mismatched or negative control patients (Figure 1).

Table 1 shows characteristics of cases and controls. Baseline characteristics, such as gender (40% female), age (median of 55 years) and ward type (mainly surgical floors) did not show statistically significant differences between cases and controls.

**Table 1:** Overview and characteristics of cases and controls out of the nested case control study

Characteristic	Case	Control	P value
n	6	225	
**Gender** = f (%)	3 (50.0)	88 (39.1)	0.908
**Age** (median [IQR])	59.5 [45.5, 70.5]	55.00 [35, 68]	0.678
**Ward type**			0.389
Intensive Care	0 (0.0)	10 (4.4)	
Intermediate Care	2 (33.3)	22 (9.8)	
Medicine	0 (0.0)	13 (5.8)	
Mixed/Diverse	0 (0.0)	6 (2.7)	
Paediatrics	1 (16.7)	21 (9.3)	
Surgery	3 (50.0)	153 (68.0)	
**Contact type**			0.001
Double room	2 (33.3)	8 (3.6)	
Multiple bed room, open wards	4 (66.7)	172 (76.4)	
Same ward	0 (0.0)	45 (20.0)	
**Exposure time (days)** (median [IQR])	6 [2.75, 8.5]	1 [0, 3]	0.016
**Delay between last contact until screening (days)** (median [IQR])	12.5 [7.25, 92.75]	58 [9, 129]	0.320

Abbreviations: MRSA. Meticillin resistant *Staphylococcus aureus,* spa. *Staphylococcus aureus* Protein A, IQR. Interquartile range.

The interval between last contact and screening was different but not statistically significant for cases (median of 12.5 days [IQR 7–93]) versus for controls (58 days [9–129], *P*=0.32).

In contrast, the type of contact for the case patients was more often a “double room” compared with the controls, where close contact had occurred less frequently (*P* = 0.001). In addition, exposure time was longer for cases (median of 6 days [IQR 3–9]) than for controls (1 day [0–3], *P*=0.016).

Characteristics of index/contact (I/C) matched pairs are described in Table 2. The main I/C matches were spa type t002 (4/6; 67%). All six I/C matches occurred in “same-room” contacts. In the three I/C matches screened within eight days after last I/C contact the exposure time was ≥5 days. Three matches were identified upon readmission and demonstrated variable exposure times.

**Table 2:** Characteristics of the six cases with Index/Contact *spa* type matched meticillin-resistant *Staphylococcus aureus*

Year	*Spa-*type Index	*Spa-*type Contact	Ward type	Spatial contact (room vs. ward)	Room type (number of beds)	Exposure time (days)	Interval between last contact and screening (days)
2012	t2231	t2231	Surgery	Room	2	5	0
2008	t002	t002	Intermediate care	Room	4–6	5	7
2008	t002	t002	Intermediate care	Room	4–6	7	8
2011	t002	t002	Surgery	Room	2	1	17^b^
2009	t005^a^	t005^a^	Paediatrics	Room	4–6	32	118^b^
2014	t002 & txAA	t002	Intermediate care	Room	4-6	1	160^b^

Abbreviations: MRSA. Meticillin resistant *Staphylococcus aureus,* spa. *Staphylococcus aureus* Protein A.

a Index/Contact were twins with continued exposure after hospitalization.

b Screened upon readmission.

## Discussion

In this study, we evaluated the MRSA contact screening strategy at Bern University Hospital, a 950 bed tertiary care hospital in Switzerland, from 2008-2014. Even though the screening strategy included an extensive number of screenings, it revealed only few I/C matches (0.2%) based on *spa*-typing. All of these patients were “same-room” contacts and had a longer median exposure time of 6 days (versus median of 1 day in the controls).

Our study had several important limitations: First, there was no universal MRSA screening at the hospital and further index cases may have been missed. Also, we did not control for potential differences based on swab localization or number of swabs per patient. We had no information on potential concurrent antibiotic therapies that could have masked an MRSA carrier status. Finally, the discriminative power of *spa*-typing is inferior to that of whole genome sequencing [9,10] and not all I/C pairs may represent transmissions given the limited resolution of *spa*-typing and/or very short exposure time.

Also, to define the time period between MRSA contact and established colonization more precisely a daily screening routine would have been required. This was not within the scope of our study and we used the available clinical data instead.

Our data suggest that screening “same-room” contacts with a specified minimal exposure time is sufficient in a low prevalence setting. This conclusion is consistent with the findings of Ng *et al.* who stated that roommates exposed for more than 48h to an MRSA index patient are at significantly greater risk of colonization than those with shorter exposure [11].

In addition, we were able to duplicate the finding from Ng *et al.* that MRSA was only detected in screenings with a certain delay after the contact: In their study, MRSA detection was better if screening occurred 3–7 days after the last contact between an MRSA index and the contact patient than on the day of exposure [11]. These findings are consistent with findings from Evison and Mühlemann [12], who suggested the optimal screening time is about 4–6 days after the last contact.

Despite the limitation of a small number of cases, our findings are likely to be generalizable to other hospitals in low prevalence settings. Importantly, we cannot make a statement on whether our screening strategy also applies to high-prevalence settings. Further studies are required to define the optimal strategy in such settings. Thus, our findings may help other hospitals in low prevalence settings implement an adequate screening strategy of MRSA contact patients.

## Conclusion

In this study, we found that the extensive ward-wide MRSA screening strategy revealed only few index/contact matches based on *spa*-typing. Our data suggest that screening “same-room” contacts with a specified minimal exposure time is sufficient in terms of identifying positive contacts in a low prevalence setting. Other elements of the strategy (such as readmission screening or “same ward” screening) yielded only very few matches. While the “same ward” screening can likely be foregone, we also believe readmission screening should not be extended beyond a certain time frame and therefore modified our screening policy so that contact patients are only traced for one year following the contact. Consequently, in our institution we modified and simplified our MRSA screening strategy according to these findings.

## Ethics approval

Given that this work was done as part of routine infection prevention activities, no approval by the Cantonal Ethics committee, Bern, Switzerland, was necessary.

## Conflict of interest statement

None.

## Funding sources

This research did not receive any specific grant from funding agencies in the public, commercial, or not-for-profit sectors.

## Authors' contributions

MB, RS, and CC had full access to all of the data in the study and take responsibility for the integrity of the data and the accuracy of the data analysis.

MB, RS, JM designed the study. MB and RS collected and analysed data, and prepared the manuscript. MK and CC performed spa-typing. RS, CC, SD and JM supervised the manuscript. RS did the statistical analysis.

All authors have reviewed and approved the manuscript. All authors have contributed significantly to the work. The manuscript has not been previously published nor is it being considered for publication elsewhere.

## Abbreviations

MRSA: Meticillin resistant Staphylococcus aureus
spa: Staphylococcus aureus Protein A

## References

[1] Tubbicke A., Hubner C., Hubner N.O., Wegner C., Kramer A., Flessa S. (2012). Cost comparison of MRSA screening and management - a decision tree analysis. BMC Health Serv Res.

[2] Collins J., Raza M., Ford M., Hall L., Brydon S., Gould F.K. (2011). Review of a three-year meticillin-resistant *Staphylococcus aureus* screening programme. J Hosp Infect.

[3] RKI (2014).

[4] Köck R., Becker K., Cookson B., van Gemert-Pijnen J.E., Harbarth S., Kluytmans J., ECDC (2014). Systematic literature analysis and review of targeted preventive measures to limit healthcare-associated infections by meticillin-resistant *Staphylococcus aureus*. Euro Surveill.

[5] Harbarth S., Hawkey P.M., Tenover F., Stefani S., Pantosti A., Struelens M.J. (2011). Update on screening and clinical diagnosis of meticillin-resistant *Staphylococcus aureus* (MRSA). Int J Antimicrob Agents.

[6] CDC (2006).

[7] Calfee D.P., Salgado C.D., Milstone A.M., Harris A.D., Kuhar D.T., Moody J. (2014). Strategies to prevent meticillin-resistant *Staphylococcus aureus* transmission and infection in acute care hospitals: 2014 update. Infect Control Hosp Epidemiol.

[8] Harmsen D., Claus H., Witte W., Rothganger J., Claus H., Turnwald D. (2003). Typing of meticillin-resistant *Staphylococcus aureus* in a university hospital setting by using novel software for spa repeat determination and database management. J Clin Microbiol.

[9] Bartels M.D., Petersen A., Worning P., Nielsen J.B., Larner-Svensson H., Johansen H.K. (2014). Comparing whole-genome sequencing with Sanger sequencing for spa typing of meticillin-resistant *Staphylococcus aureus*. J Clin Microbiol.

[10] Durand G., Javerliat F., Bes M., Veyrieras J.B., Guigon G., Mugnier N. (2018). Routine Whole-Genome Sequencing for Outbreak Investigations of *Staphylococcus aureus* in a National Reference Center. Front Microbiol.

[11] Ng W., George K., Muhammed N., Tomassi J., Katz K.C. (2012). Too close for comfort: screening strategy to detect meticillin-resistant *Staphylococcus aureus* conversion in exposed roommates. Am J Infect Control.

[12] Evison J., Muhlemann K. (2008). Screening for carriage of meticillin-resistant *Staphylococcus aureus* shortly after exposure may lead to false-negative results. Infect Control Hosp Epidemiol.

